# Immunoendocrine Profiles in Neurocysticercosis Patients: A Case-Control Study in Honduras

**DOI:** 10.3390/tropicalmed11020051

**Published:** 2026-02-12

**Authors:** Nicholas Zugno-Gadea, Lázaro Molina, Mariangela Hernandez-González, María Mercedes Rueda, Francis Bejarano, Nelson Alexander Betancourt, Ana Sanchez

**Affiliations:** 1 Department of Health Sciences, Brock University, Niagara Region, 1812 Sir Isaac Brock Way, St. Catharines, ON L2S 3A1, Canada; ngadea@brocku.ca; 2 Neurology Department, Hospital Escuela Universitario, Tegucigalpa 11101, Honduras; neurologia1999@gmail.com (L.M.); fbshigure1986@gmail.com (F.B.); tiroglobulina13@gmail.com (N.A.B.); 3 Faculty of Math and Sciences, Brock University, Niagara Region, 1812 Sir Isaac Brock Way, St. Catharines, ON L2S 3A1, Canada; mh22ue@brocku.ca; 4 Department of Parasitology, School of Microbiology, National Autonomous University of Honduras, Tegucigalpa 11101, Honduras; maria.rueda@unah.edu.hn

**Keywords:** neurocysticercosis, *Taenia solium*, neglected tropical diseases, biomarkers, immunoendocrine response, Honduras

## Abstract

Emerging evidence suggests that certain cestodes, including *Taenia solium*, may actively modulate the host’s hormonal and immune environment to facilitate their survival. This study aimed to determine whether patients diagnosed with neurocysticercosis (NCC) exhibit immunoendocrine alterations associated with infection. A clinical study was conducted in Honduras, enrolling 11 adult NCC patients (9 female, 2 male) and 11 age- and sex-matched healthy controls. Serum concentrations of seven hormones and two cytokines were evaluated. Compared to controls, NCC patients showed significantly elevated levels of 17β-Estradiol (E2), Progesterone (P4), Androstenedione (A4), Luteinizing Hormone (LH), Follicle-Stimulating Hormone (FSH), Interleukin-6 (IL-6), and Interleukin-10 (IL-10). Conversely, Free testosterone (FT) and Dihydrotestosterone (DHT) levels were significantly reduced. These findings support the hypothesis that *T. solium* may manipulate host immunoendocrine pathways to promote its establishment and persistence within the central nervous system.

## 1. Introduction

Neurocysticercosis (NCC) is a condition characterized by the infiltration and localization of the metacestode (cysticercus) stage of the tapeworm parasite *Taenia solium* within the central nervous system (CNS) [1].

The host–parasite interaction in *Taenia solium* NCC remains incompletely defined and continues to be investigated extensively [2,3]. Clinical severity is attributed to several factors including the localization of cysticerci within the CNS, parasite load (the number of parasitizing cysticerci), and the intensity of inflammatory reactions by the host. Recent evidence suggests that helminthic larval stages may directly contribute to neuropathology by inducing caspase 9-mediated mitochondrial dysfunction and cellular apoptosis within host tissues. Such intrinsic apoptotic signaling could amplify neuroinflammatory cascades, particularly in cases of high parasite burden or prolonged cyst viability [4]. Biological sex of the patient may also be associated with disease severity; compared to men, women seem to provide a more favorable hormonal environment for the parasite to grow [5,6]. Several reports suggest that the proportion of damaging cysticerci are significantly higher in women than in men and that women experience more severe clinical presentations caused by multiple degenerating cysticerci within the brain parenchyma [5,6,7]. Due to these sex-associated differences, the hormonal profile of the host has been suggested to play a large role in the viability of the parasite. For instance, in pigs, cysticercosis prevalence increases significantly when male pigs are castrated (resulting in impaired testosterone production) [8], or when female pigs are pregnant (characterized by enhanced estrogen production) [9]. Further, in chronically infected hosts, the parasite appears to modify its hormonal environment by converting testosterone (FT) into 17β-Estradiol (E2) [9]. In brief, evidence suggests that estrogens, such as E2, increase parasite loads; androgens, such as FT, decrease them in two ways: (i) by acting directly on the parasite, favoring or hindering its reproduction; and (ii) by biasing the immune response of the host toward a parasite-permissive instead of a parasite-restrictive response (Th2 and Th1, respectively) [10,11]. Further, the finding that *T. solium* is also able to synthesize steroid hormones adds a new layer of complexity in understanding host–parasite interactions [9,12,13,14].

Exploration of how the endocrine system of a host can, under certain circumstances, favor or hinder the establishment of cysticerci, can lead to further understanding of the immunoendocrine interactions at play in the host–parasite relationship. The aim of the present work was, therefore, to investigate whether the presence of the *T. solium* cysticerci in the human CNS is associated with concentrations of specific sex hormones and other immune biomarkers

## 2. Methods and Patients

This study was designed as a case-control analysis to determine whether neurocysticercosis (NCC) is associated with distinct immunoendocrine profiles. The design allowed comparison of cytokine and hormone concentrations between imaging confirmed NCC patients and age- and sex-matched healthy controls.

### 2.1. Study Area and Community

In collaboration with the National Autonomous University of Honduras (UNAH), the study was conducted in Tegucigalpa. NCC patients were recruited at the university’s medical school and teaching hospital, Hospital Escuela Universitario (HEU). Healthy controls were recruited at UNAH’s main campus, Ciudad Universitaria.

### 2.2. Study Population and Recruitment

Eligible NCC patients were Honduran adults attending neurology consultations at HEU who met the following criteria:

(i) age 16–61 years;
(ii) born and raised in Honduras;
(iii) past or present definitive diagnosis of NCC according to international guidelines;
(iv) absence of chronic neurological, hormonal, or immunosuppressive conditions.

Healthy controls were Honduran adults of similar age and sex who had:

(i) no past or present diagnosis of cysticercosis;
(ii) no chronic neurological, hormonal, or immunosuppressive conditions.

Exclusion criteria for both groups included pregnancy, use of medications affecting hormone or cytokine levels (e.g., hormonal contraception), menopause, prepubescence, travel outside Honduras within the five years preceding NCC diagnosis, and daily contact with individuals with taeniasis.

### 2.3. Participant Characteristics

A total of 11 NCC patients were enrolled. Of these, 9 (81.8%) were female (age range 16–61 years; mean ± SD: 34.2 ± 13.8) and 2 (18.2%) were male (ages 36 and 51 years; mean ± SD: 43.5 ± 10.6). Each patient was matched with a healthy control based on age and sex (control age range 20–60 years; mean ± SD: 37.9 ± 8.1). No significant age difference was observed between groups (*p* = 0.19). All participants met inclusion criteria; the initial planned age range (18–45 years) was expanded to 16–61 years due to recruitment constraints. Because only two male participants were included on the cohort, the study was not designed or powered to evaluate sex-based differences. Therefore, analysis was conducted on the overall sample, and no statistical comparison between men and women was performed.

### 2.4. Data Collection

Clinical, demographic, and epidemiological data were collected by the neurologist during patient appointments following informed consent. Healthy volunteers were screened and enrolled by trained study personnel at UNAH. Venous blood samples (10 mL) were collected from all participants and stored at −20 °C until transport to Canada for laboratory analysis.

### 2.5. Laboratory Analyses

The following analytes were measured:

(a) Cytokines (Interleukin-6 (IL-6) and Interleukin-10 (IL-10);
(b) Gonadotropins (Pituitary Hormones): Luteinizing Hormone (LH) and Follicle-Stimulating Hormone (FSH);
(c) Steroid hormones and precursors: A4 (Androstenedione), P4 (Progesterone), E2 (17β-Estradiol), FT (Free testosterone), and DHT (Dihydrotestosterone);
(d) Circulating antibodies to purified glycoprotein antigens derived from *T. solium* cysticerci.

IL-6 and IL-10 were measured with ELISAs as per manufacturer instructions from Millipore Sigma.

Hormones LH, FSH, A4, P4, E2, T4, and DHT were measured with ELISA’s as per manufacturer instructions from Diagnostics Biochem Canada Inc.

Anti-*T. solium* antibodies were detected using the Cysticercosis IgG Western blot (WB) assay (LDBIO Diagnostics, France). In this assay, cysticercal glycoproteins are separated by electrophoresis, transferred onto a nitrocellulose membrane, and distributed according to their molecular weights. Specific IgG antibodies in the samples bind to these antigens and appear as purple transverse bands. Although positive samples may show multiple bands between 2 and 200 kilodaltons (kDa), only those within the 6–55 kDa range are considered for interpretation due to their higher specificity. The bands most commonly observed fall at approximately 50–55, 39, 23–26, 12, and 6–8 kDa. According to the manufacturer, the presence of a minimum of 2 well defined bands is indicative of cysticercosis.

### 2.6. Imaging

All patients had a confirmed diagnosis of NCC based on prior brain imaging, meaning each had already undergone CT or MRI evaluation before enrollment.

### 2.7. Statistical Analysis

Analyte data were measured against known reference ranges (if appropriate), and when possible, matching was performed by age and sex. Data was recorded in Excel and analyzed using SAS (v. 9.4). Fisher’s Exact test was conducted on each participant pair (patient and control) to determine degree of matching. Wilcoxon Ranked Sum test was conducted to compare the difference in analyte levels between patients and controls. Both tests are two-tailed in nature, and differences were considered statistically significant at *p* ≤ 0.05. Detailed outputs from the Wilcoxon rank-sum test for all cytokines and hormones are presented in Supplementary File. Descriptive summaries are presented as medians with interquartile ranges and data ranges using boxplots. Given the small sample size and heterogeneity in cyst stage and clinical presentation, the study was not powered to assess correlations between immunoendocrine markers and disease burden or symptom severity

### 2.8. Ethics Considerations

The study received ethical approval from participating institutions, both in Canada and Honduras (Brock University. File number #18-046; UNAH, File number # 003-2019—CEIC/HEU).

## 3. Results

### 3.1. Clinical Presentation of Patients

As presented in Table 1, all 11 NCC patients diagnosed exhibited epilepsy, with convulsive episodes observed in 10 cases (90.9%) The predominant seizure types included tonic–clonic seizures (18.2%), secondary generalized seizures (18.2%), and complex focal seizures (18.2%). Eight patients (72.7%) reported chronic headaches or migraines. Additional neurological manifestations included hydrocephalus in 18.2% (*n* = 2) and papilledema in 9.1% (*n* = 1). Other clinical features were somatosensory deficits (18.2%), movement disorders (27.3%), hemiparesis (18.2%), Parinaud’s syndrome (9.1%), short-term memory impairment (9.1%), cognitive decline (9.1%), and meningeal involvement (9.1%).

### 3.2. Cysticerci Localization

As seen in Table 2, eight of the eleven patients (72.7%) presented parenchymal cysticerci location, whilst the remaining three (27.3%) demonstrated extra parenchymal involvement. In terms of disease activity, 8 patients (72.7%) were categorized as active NCC, whereas 3 patients (27.3%) were classed as inactive. Imaging staging revealed that 3 patients had vesicular cysts, 3 had colloidal cysts, 3 presented with granular or calcified lesions, and 2 displayed mixed phases.

### 3.3. Serological Findings: Anti-T. solium Antibodies

**Figure 1:**
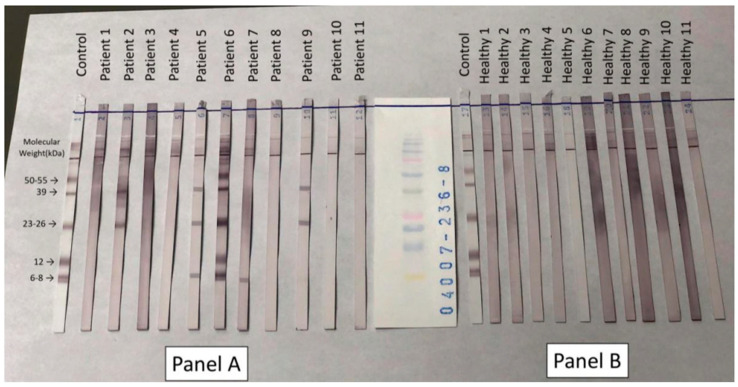
Anti-*T. solium* IgG reactivity through Western blot analysis of patients (**Panel A**) and controls (**Panel B**). In (**Panel A**), patients 2, 5, 6, 7, and 9 were positive for specific anti-*T. solium* antibodies as shown by their reactivity to two or more diagnostic glycoproteins. The remaining six patients did not present detectable antibody responses. No participants in the healthy control group presented reactivity to any of the seven glycoproteins.

As shown in Figure 1, Western blot analysis for anti-*T. solium* antibodies revealed no detectable reactivity to any glycoprotein bands in the healthy controls. In contrast, circulating anti-*T. solium* IgG antibodies were detected in 5 of the 11 NCC patients (45.5%; patients 2, 5, 6, 7, and 9). Two or more bands were visible in all positive samples; however, in Figure 1, in the sample from patient 7 only one band in the 6–8 kDa region is visible. A faint band in the 23–26 kDa region was perceptible to the naked eye under different lighting and viewing angles, but it was not captured in the digital image; for transparency, we did not enhance or modify the figure.

### 3.4. Immunoendocrine Profiles

Due to the small sample size, medians instead of means were calculated to compare analyte concentrations. Below, analyte concentrations are presented with detailed Wilcoxon test statistics in the text (Z values, *p*-values, median ranks), complemented by boxplots that visually illustrate the distributional differences between groups. For all analytes reported, tables containing Wilcoxon test statistics can be found in Supplementary File (Tables S1–S6). Table 2 contains individual data for each NCC patient as well as the concentration of their studied analytes. It is worth noting that patient four (female, 61) and six (Male, 36) were mother and son. Even though this could indicate a common source of infection, epidemiological data did not include this information.

Table 3 presents the values of nine immunoendocrine markers categorized by group (patients versus controls).

### 3.5. Cytokines (IL-6 and IL-10)

**Figure 2:**
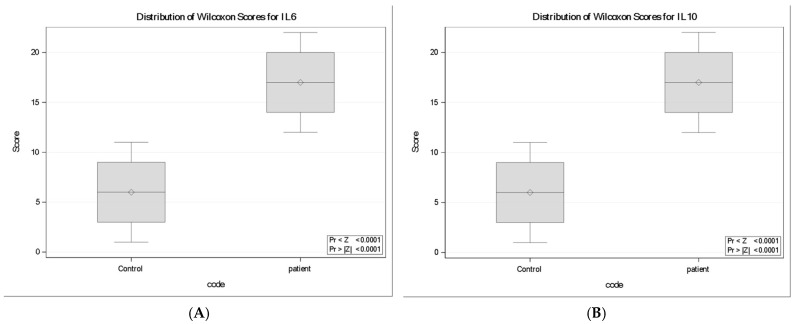
Distribution of Wilcoxon rank scores for (**A**) IL-6 and (**B**) IL-10 in controls and neurocysticercosis patients (*n* = 11 per group). Boxplots display the median rank (horizontal line), interquartile range (box), and range (whiskers); diamonds indicate the mean rank. Rank scores were significantly higher in patients for both cytokines (IL-10: Z = −3.94, *p* < 0.0001; IL-6: Z = −3.94, *p* < 0.0001), consistent with an upward shift of the patient distributions relative to controls.

IL-10 concentrations were significantly higher in neurocysticercosis patients compared with controls (Wilcoxon two-sample test: Z = −3.94, *p* < 0.0001), with mean rank scores of 17.0 in patients versus 6.0 in controls. Similarly, IL-6 concentrations were significantly elevated in patients (Z = −3.94, *p* < 0.0001), with mean ranks of 17.0 compared with 6.0 in controls. Boxplots (Figure 2) corroborated these findings, showing upward shifts in the rank distributions of both IL-10 and IL-6 in infected individuals relative to controls.

### 3.6. Progesterone (P4)

**Figure 3:**
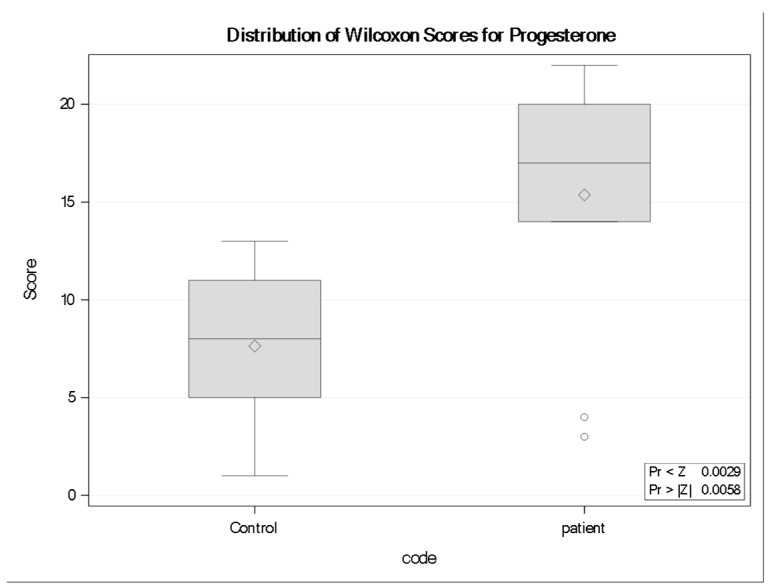
Distribution of Wilcoxon rank scores for Progesterone (P4) in controls and neurocysticercosis patients (*n* = 11 per group). Boxplots display the median rank (horizontal line), interquartile range (box), and range (whiskers); diamonds indicate the mean rank. Patients showed higher rank distributions than controls (Wilcoxon rank-sum test: Z = −2.758, two-sided *p* = 0.0058; one-sided Pr < Z = 0.0029). Two low-rank outliers are present in the patient group, increasing within-group variability but not altering the overall direction or significance of the difference.

Progesterone (P4) concentrations were significantly higher in neurocysticercosis patients than in controls. Wilcoxon rank-sum analysis demonstrated a statistically significant difference between groups (Z = −2.758, *p* = 0.0058), with patients exhibiting a higher rank distribution (mean rank = 14.00) compared with controls (mean rank = 7.64). Boxplot visualization supported this finding, showing an overall upward shift in P4 values among infected individuals (Figure 3). Although two patient values appeared as low outliers, contributing to a wider distribution within the patient group, these observations did not negate the overall between-group difference.

### 3.7. Androstenedione (A4)

**Figure 4:**
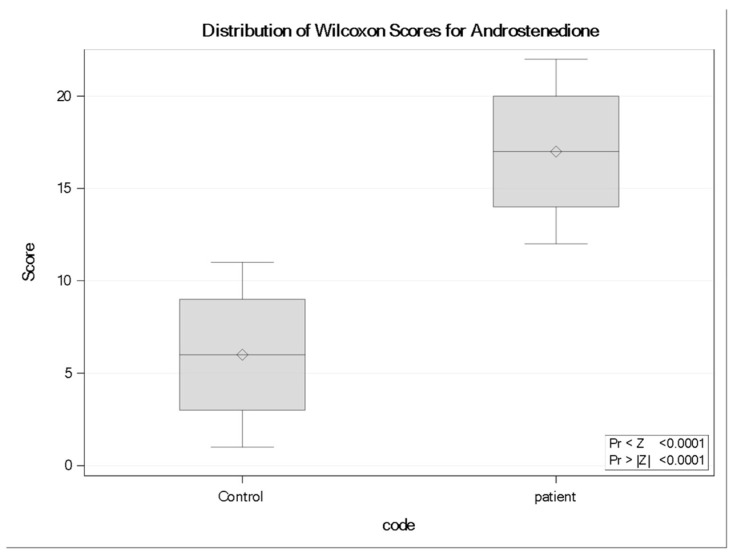
Distribution of Wilcoxon rank scores for Androstenedione (A4) in controls and neurocysticercosis patients (*n* = 11 per group). Boxplots display the median rank (horizontal line), interquartile range (box), and range (whiskers); diamonds indicate the mean rank. Patients showed higher rank distributions than controls (Wilcoxon rank-sum test: Z = −3.9399, two-sided *p* < 0.0001).

Concentrations of the hormone precursor of androgens and estrogens, Androstenedione (A4), were significantly elevated in neurocysticercosis patients compared to controls. The Wilcoxon two-sample test revealed a strong statistical difference (Z = −3.9399, *p* < 0.0001), with patients exhibiting a higher rank distribution (mean rank = 17.0) compared with controls (mean rank = 6.0). Boxplot analysis confirmed this elevation, showing a consistent upward shift in A4 levels among infected individuals (Figure 4).

### 3.8. Dihydrotestosterone (DHT)

**Figure 5:**
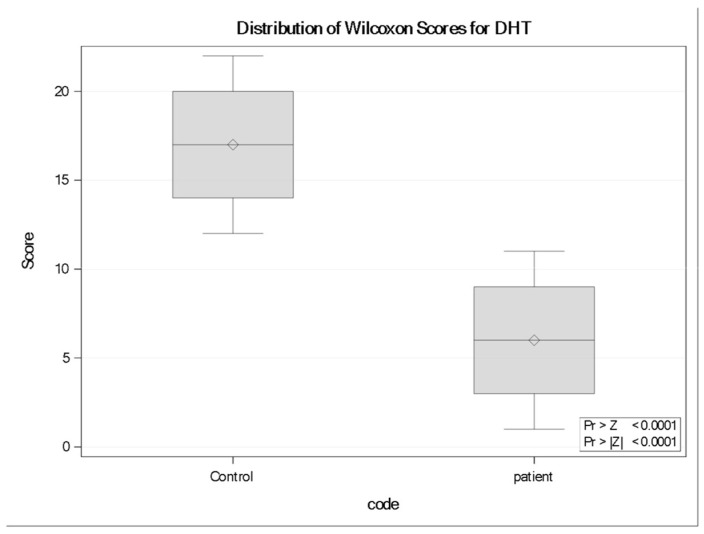
Distribution of Wilcoxon rank scores for Dihydrotestosterone (DHT) in controls and neurocysticercosis patients (*n* = 11 per group). Boxplots display the median rank (horizontal line), interquartile range (box), and range (whiskers); diamonds indicate the mean rank. Controls exhibited significantly higher rank distributions than patients (Wilcoxon rank-sum test: Z = +3.9399, two-sided *p* < 0.0001).

Dihydrotestosterone (DHT) values were significantly higher in controls than in neurocysticercosis patients (Wilcoxon rank-sum test: Z = +3.9399Z, two-sided *p* < 0.0001); controls exhibited higher rank distributions (mean rank = 17.0) compared with patients (mean rank = 6.0). Boxplot analysis confirmed this reduction, revealing a consistent downward shift in DHT levels among infected individuals (Figure 5).

### 3.9. Gonadotropins (LH and FSH)

**Figure 6:**
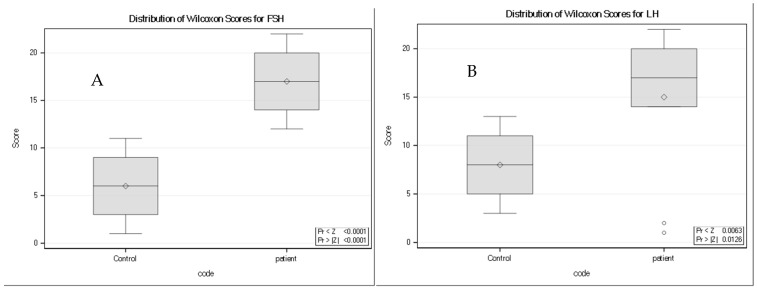
Distribution of Wilcoxon rank scores for (**A**) Follicle-Stimulating Hormone (FSH) and (**B**) Luteinizing Hormone (LH) in controls and neurocysticercosis patients (*n* = 11 per group). Boxplots display the median rank (horizontal line), interquartile range (box), and range (whiskers); diamonds indicate the mean rank. For both gonadotropins, patients exhibited higher rank distributions than controls. Group differences were evaluated using the Wilcoxon rank-sum test with tie correction where applicable (FSH: Z = −3.9410, two-sided *p* < 0.0001; LH: Z = −2.4953, two-sided *p* = 0.0126).

Gonadotropin levels differed significantly between groups. Both Luteinizing Hormone (LH) and Follicle-Stimulating Hormone (FSH) showed higher rank distributions in neurocysticercosis patients compared with controls (Figure 6). Wilcoxon rank-sum analysis demonstrated a significant between-group difference for FSH (Z = −3.9410, two-sided *p* < 0.0001, and a statistically significant difference for LH (Z = −2.4953, two-sided *p* = 0.0126).

### 3.10. Free Testosterone (FT) and Estradiol (E2)

**Figure 7:**
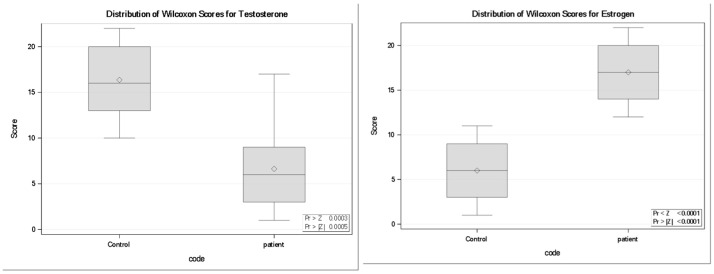
Distribution of Wilcoxon rank scores for Free Testosterone and Estradiol levels in controls and neurocysticercosis patients (*n *= 11 per group). Boxplots display the median rank (horizontal line), interquartile range (box), and range (whiskers); diamonds indicate the mean rank. FT ranks were significantly lower in patients compared with controls (Wilcoxon two-sample test, Z = 3.48, *p* = 0.0005), whereas E2 ranks were significantly higher in patients (Z = −3.94, *p* < 0.0001). Together, the boxplots illustrate a downward shift in FT and a consistently elevated distribution of E2 among infected individuals relative to controls.

Free testosterone (FT) concentrations were significantly reduced in neurocysticercosis patients compared with controls. The Wilcoxon two-sample test indicated a strong group difference (Z = 3.48, *p* = 0.0005), with patients exhibiting substantially lower mean rank scores than controls (6.64 vs. 16.36). In contrast, Estradiol (E2) levels were significantly elevated in patients (Z = −3.94, *p* < 0.0001), with a higher mean rank in patients (17.0) compared with controls (6.0). Boxplot analyses (Figure 7) corroborated these findings, demonstrating a downward shift in FT and a consistently higher distribution of E2 among infected individuals relative to controls.

## 4. Discussion

This study examined whether the presence of *T. solium* cysticerci in the CNS is associated with distinct alterations in host immunoendocrine profiles. By integrating cytokine and hormone measurements, we identified patterns suggestive of a coordinated shift in both inflammatory and endocrine pathways, that translates into an estrogen-dominant, Th2-skewed immunological milieu, consistent with observations from animal studies that demonstrate estrogens promote cysticerci proliferation while androgens inhibit parasite viability [15,16]. Although sex-based differences in immunoendocrine responses are biologically plausible, the study included only two male participants, precluding meaningful sex-stratified analyses or visual representation of sex-specific patterns. Larger, sex-balanced cohorts will be required to evaluate potential sex-specific immunoendocrine profiles.

The present findings suggest that in humans NCC may be accompanied by systemic immunoendocrine changes with potential relevance for host–parasite interactions. Recent transcriptome studies on *T. solium* subarachnoid cysticerci have identified molecular pathways associated with survival and host interaction, indicating the parasite’s ability to adapt to and possibly exploit human hormonal cues [17,18]. Our findings are also supported by older animal models aiming to determine the effect of various hormones on cysticercosis outcomes. For example, murine studies of *T. crassiceps* infection consistently show that females carry higher parasite burdens than males, a disparity abolished by male gonadectomy [19,20]^.^ Similarly, other authors have shown that administration of 17β-Estradiol experimentally enhances parasite survival and burden in infected male mice [21]. Based on the *T. crassiceps* murine model, Larralde and collaborators (1995) determined that cysticercosis can cause significant hormonal changes in male hosts, including a 200-fold increase in estrogen synthesis and a 90% decrease in testosterone production [22]. Parallel findings in porcine models of *T. solium* infection indicate notable testosterone suppression in infected boars [11], alongside heightened prevalence and parasite load in castrated or pregnant pigs [23].

Patients exhibited elevated IL-6 and IL-10, reflecting a modified inflammatory profile. IL-6 is a pleiotropic pro-inflammatory cytokine that promotes acute-phase responses, B-cell differentiation, leukocyte recruitment, astrocyte activation, and increased blood–brain barrier permeability, all of which contribute to the inflammatory reaction surrounding degenerating cysticerci [24,25,26,27,28]. In contrast, IL-10 is a key immunoregulatory mediator that limits excessive Th1-driven inflammation [29]. The coexistence of elevated IL-6 and IL-10 aligns with the mixed Th1/Th2 immune response previously described in chronic NCC [30,31,32]. IL-6 can also stimulate IL-10 production, creating a regulatory feedback loop that may facilitate parasite persistence within the CNS [33]. These cytokine patterns may also intersect with the hormonal profiles observed in our study, as sex steroids such as Estradiol and Progesterone are known to modulate IL-6 and IL-10 production through both genomic and non-genomic pathways. Although our sample size does not allow sex-specific analyses, the overall profile supports the concept of a bidirectional interaction between endocrine and immune responses during chronic infection.

In addition, Progesterone and Estradiol concentrations were higher in patients, consistent with a hormonally immunosuppressive milieu. It is widely known that progesterone enhances IL-10 production, largely through the progesterone-induced blocking factor pathway, thereby contributing to an anti-inflammatory milieu that supports immune regulation [34,35].

Notably, two patient values appeared as low outliers for Progesterone, falling below the lower range of both groups. These values did not alter the statistical significance of the group difference but highlight biological heterogeneity that may reflect sex, reproductive status, or other individual factors. Although the sample size restricts conclusive findings, these results correspond with previous literature indicating increased progesterone levels in NCC [36].

In contrast, testosterone and DHT were reduced in patients. Given that androgens can suppress pro-inflammatory cytokine production, their depletion may contribute to a more permissive inflammatory state. Androgens such as testosterone and DHT have been shown to suppress pro-inflammatory cytokine production of TNF-α, IL-1β, and IL-6 through androgen receptor–mediated modulation of immune cell function [37,38,39].

Luteinizing Hormone (LH) and Follicle-Stimulating Hormone (FSH) levels were significantly higher in patients with NCC compared to controls, suggesting compensatory activation of the hypothalamic–pituitary axis in response to peripheral hormonal disruption, although the precise mechanisms remain unclear. These findings are consistent with previous investiagtions reporting that gonadotropins may contribute to parasite–host interactions during *T. solium* infection. As it is widely known, elevated LH stimulates theca cell production of Androstenedione, a precursor for estrogen synthesis [40], while increased FSH enhances aromatase activity, a key enzyme in the deandrogenization process that is particularly relevant under inflammatory conditions [40,41,42]. In our cohort, the lower LH values observed in the two male patients suggest that the marked LH elevation may be more pronounced in females, whereas FSH increases were consistent across sexes.

Prior studies have similarly reported increased LH in male NCC patients, alongside elevated FSH and reduced testosterone in clinically severe cases [43]. Experimental models of chronic infection further support a role for FSH as a mediator of estrogen production [43]. Human chorionic gonadotropin (hCG), which shares a common α-subunit with LH and, to a lesser extent, with FSH, exerts comparable pharmacological actions [44]. hCG has been shown to stimulate estrogen production in the Leydig cells of both sexes [8,44], and to promote parasite development, as in vitro studies demonstrate enhanced reproduction and maturation of *T. solium* and *T. crassiceps* cysticerci in its presence [45]. The elevated gonadotropin concentrations observed in NCC may not only alter host steroid hormone balance but also act as mitogenic signals that facilitate parasite growth and reproduction, underscoring the complex endocrine–parasitic interplay in this disease.

As a whole, these findings suggest an immunoendocrine reprogramming in neurocysticercosis, characterized by a mixed cytokine profile alongside alterations in sex steroid and gonadotropin levels. Experimental work in murine cysticercosis has demonstrated that hypothalamic–pituitary–gonadal axis hormones modulate immune responses, supporting the plausibility of systemic endocrine involvement during parasite infection [46]. More broadly, reviews of neuroendocrine–immune interactions emphasize the bidirectional communication between sex steroids, cytokines, and neural circuits, reinforcing the interpretive framework for the associations observed here [47].

Finally, it is worth mentioning that only 45.5% of NCC patients exhibited circulating antibodies by Western blot analysis, a detection rate that aligns with the variable sensitivity of serum-based EITB testing documented in prior studies [48,49]. The observed proportion of seropositive patients indicates the established limitations of serum-based antibody detection in NCC. Sanchez et at. in 1999 observed a significant decrease in the sensitivity of EITB in serum relative to cerebrospinal fluid (CSF), especially in instances involving calcified lesions, with only 28% of patients yielding positive results [49]. The findings can be attributed to two mechanisms: (i) intrathecally produced antibodies frequently do not cross the blood–brain barrier in detectable concentrations, and (ii) antigenic stimulation decreases as cysticerci degenerate and calcify, leading to antibody levels falling below assay detection thresholds. This may explain seronegativity in NCC confirmed cases, particularly in instances of single-lesion infections or chronic disease stages [48].

Among the study limitations, first, it is important to mention the cross-sectional design and modest sample size, which naturally limit causal inference and generalizability. For the same reason, the study was not powered to evaluate correlations between immunoendocrine markers and disease burden or symptom severity; such analyses would risk unstable or misleading inferences in a cohort of this size and heterogeneity. Individual variability, including sex, age, reproductive status, and unknown comorbidities, may also influence hormone levels and should be considered in future studies. Secondly, our study was not powered to assess such differences due to the very small number of male participants; they were included solely to reflect the available patient population and did not influence the distribution or interpretation of the overall results. Thirdly, because menstrual cycle phase was not recorded, cycle-specific hormonal variation could not be assessed. This limitation is consistent with the exploratory nature of the study and does not affect the overall interpretation of group-level hormone patterns. Finally, while cerebrospinal fluid-based WB testing may have offered enhanced sensitivity in our study, the lumbar puncture was considered unnecessary and ethically unjustifiable, as all patients had already received confirmed NCC diagnoses through imaging and clinical criteria. This decision is consistent with best clinical practices aimed at avoiding invasive procedures that do not influence patient management [50].

## 5. Conclusions

In summary, this study highlights immunoendocrine alterations associated with neurocysticercosis, suggesting that profiling hormone and cytokine patterns may provide clinically relevant insights into disease variability, patient stratification, and potential adjunctive targets for future therapeutic or diagnostic approaches.

## Figures and Tables

**Table 1:** Clinical descriptions of NCC patients.

	Female (*n* = 9)	Male (*n* = 2)	Total Patients (*n* = 11)
	# (%)	# (%)	# (%)
Age (mean ± SD)	34.2 ± 13.8 (18.8)	43.5 ± 10.6 (18.2)	35.9 ± 13.3 (100.0)
Epilepsy	9 (100)	2 (100.0)	11 (100.0)
with convulsions	8 (88.9)	2 (100.0)	10 (90.9)
without convulsions	1 (11.1)	0 (0.0)	1 (9.1)
Tonic–clonic seizures (generalized)	1 (11.1)	1 (50.0)	2 (18.2)
Simple focal seizures (partial)	1 (11.1)	0 (0.0)	1 (9.1)
Secondary generalized seizures	2 (22.2)	0 (0.0)	2 (18.2)
Complex focal seizures (partial)	1 (11.1)	1 (50.0)	2 (18.2)
Chronic headache/migraine	6 (66.7)	2 (100.0)	8 (72.7)
Short-term memory loss	1 (11.1)	0 (0.0)	1 (9.1)
Intellectual decline	1 (11.1)	0 (0.0)	1 (9.1)
Meningeal involvement	0 (0)	1 (50.0)	1 (9.1)
Somatosensory decline	2 (22.2)	0 (0.0)	2 (18.2)
Movement disorder	2 (22.2)	1 (50.0)	3 (27.3)
Hemiparesis	2 (22.2)	0 (0.0)	2 (18.2)
Hydrocephalus	2 (22.2)	0 (0.0)	2 (18.2)
Parinaud’s syndrome	1 (11.1)	0 (0.0)	1 (9.1)
Papilledema	1 (11.1)	0 (0.0)	1 (9.1)

**Table 2:** Clinical, serological, cytokine, and hormonal findings of 11 patients.

Patient Number	Sex	Age	Type of NCC at Recruitment (Active/Inactive)	Localization of Cysts	WB Results # of Bands	IL-6 (pg/mL)	IL-10 (pg/mL)	P4 (ng/mL)	FT (ng/mL)	A4 (ng/mL)	DHT (pg/mL)	E2 (pg/mL)	LH (IU/L)	FSH (IU/L)
1	F	33	Inactive (Calcified)	Parenchymal	0	85.82 ^§^	162.30 ^§^	56.46	0.25	4.30 ^§^	26.11 ^‡^	1994.14 ^§^	89.35 ^§^	78.41 ^§^
2	F	16	Active (Colloidal)	Extraparenchymal	2	89.74 ^§^	425.84 ^§^	59.22	0.29	5.70 ^§^	27.59 ^‡^	2140.11 ^§^	94.65 ^§^	67.68 ^§^
3	F	47	Active (Colloidal)	Parenchymal	0	255.63 ^§^	373.37 ^§^	46.33	0.24	3.22 ^§^	26.51 ^‡^	984.71 ^§^	85.45 ^§^	57.31 ^§^
4	F *	61	Inactive (Calcified)	Parenchymal	0	42.86 ^§^	285.37 ^§^	45.41	0.19 ^‡^	2.56 ^§^	34.83 ^‡^	1720.42 ^§^	94.22 ^§^	51.12 ^§^
5	M	51	Active (Colloidal)	Parenchymal	3	29.53 ^§^	164.63 ^§^	1.64	0.07 ^‡^	3.53 ^§^	22.43 ^‡^	3196.24 ^§^	2.41	49.17 ^§^
6	M *	36	Active (Vesicular)	Extraparenchymal	5	35.08 ^§^	174.37 ^§^	1.84	0.09 ^‡^	6.31 ^§^	25.35 ^‡^	3091.78 ^§^	3.61	47.89 ^§^
7	F	41	Inactive (Calcified)	Parenchymal	1	45.64 ^§^	283.62 ^§^	54.77	0.93	5.44 ^§^	34.22 ^‡^	2790.73 ^§^	85.63 ^§^	59.87 ^§^
8	F	21	Active (Colloidal)	Parenchymal	0	98.42 ^§^	378.90 ^§^	49.73	0.21	4.03 ^§^	37.32 ^‡^	973.64 ^§^	88.29 ^§^	65.09 ^§^
9	F	26	Active (Colloidal)	Extraparenchymal	2	99.75 ^§^	452.00 ^§^	53.84	0.16 ^‡^	3.39 ^§^	29.30 ^‡^	1020.33 ^§^	89.47 ^§^	78.74 ^§^
10	F	31	Active (Vesicular)	Parenchymal	0	85.37 ^§^	280.32 ^§^	59.42	0.27	3.95 ^§^	30.01 ^‡^	1162.77 ^§^	97.27 ^§^	64.89 ^§^
11	F	32	Active (Vesicular)	Parenchymal	0	57.36 ^§^	386.80 ^§^	42.20	0.36	3.88 ^§^	35.32 ^‡^	750.17 ^§^	77.49 ^§^	89.42 ^§^

NCC = Neurocysticercosis; WB = Western blot; IL = Interleukin; P4 = Progesterone. FT = Testosterone. A4 = Androstenedione. DHT = Dihydrotestosterone. E2 = Estradiol; LH = Luteinizing Hormone; FSH = Follicle-Stimulating Hormone. § = higher than reference value. ‡ = lower than reference value. * Mother and son.

**Table 3:** Median analyte values by participant group.

Analyte	Patient	Control	*p*-Value
IL-6 (pg/mL)	85.37	12.58	<0.0001 ^§^
IL-10 (pg/mL)	285.37	8.45	<0.0001 ^§^
P4 (ng/mL)	49.73	18.62	0.0058 ^§^
FT (ng/mL)	0.24	0.87	0.0005 ^§^
A4 (ng/mL)	3.95	1.84	<0.0001 ^§^
DHT (pg/mL)	29.30	55.45	<0.0001 ^§^
E2 (pg/mL)	1720.42	211.35	<0.0001 ^§^
LH (IU/L)	88.29	34.62	0.0126 ^§^
FSH (IU/L)	64.89	10.25	<0.0001 ^§^

IL = Interleukin; P4 = Progesterone. FT = Free testosterone. A4 = Androstenedione. DHT = Dihydrotestosterone. E2 = Estradiol; LH = Luteinizing Hormone; FSH = Follicle-Stimulating Hormone. § = statistically significant *p*-value.

## Data Availability

All data generated or analyzed during the study are included in this article and its Supplementary Information Files.

## Supplementary Materials

The following supporting information can be downloaded at https://www.mdpi.com/article/10.3390/tropicalmed11020051/s1, Tables S1–S6: Supplementary Material to: Immunoendocrine profiles in neurocysticercosis patients: a clinical study in Honduras.

## Abbreviations

The following abbreviations are used in this manuscript:

A4	Androstenedione
CNS	Central Nervous System
DHT	Dihydrotestosterone
E2	17β-Estradiol
EITB	Enzyme-Linked Immunoelectrotransfer Blot
ELISA	Enzyme-Linked Immunosorbent Assay
FSH	Follicle-Stimulating Hormone
HEU	Hospital Escuela Universitario (University Teaching Hospital)
IL	Interleukin
LH	Luteinizing Hormone
NCC	Neurocysticercosis
P4	Progesterone
FT	Free Testosterone
Th1	Type 1 T-helper Cell
Th2	Type 2 T-helper Cell
TNF-α	Tumor necrosis factor-alpha
UNAH	Universidad Nacional Autónoma de Honduras
WB	Western Blot
