# Supplementary material for: Immunoendocrine Profiles in Neurocysticercosis Patients: A Case-Control Study in Honduras
Source: Trop Med Infect Dis. 2026 Feb 12;11(2):51. doi: 10.3390/tropicalmed11020051 (PMC12944953; doi:10.3390/tropicalmed11020051)
Supplement: Supplementary file 1 [file tropicalmed-11-00051-s001.zip › tropicalmed-4004353-supplementary.pdf]

**TITLE**: Supplementary Material to: **Immunoendocrine Profiles in Neurocysticercosis Patients: A Case-Control Study in Honduras**

**TABLE OF CONTENTS**

**INTRODUCTION**

This Supplementary Material presents the comprehensive statistical analyses that support the conclusions stated in the main publication. It specifically presents the outcomes of the Wilcoxon Rank Sum tests that compare cytokine and hormone concentrations between neurocysticercosis (NCC) patients and healthy controls. This data supplements the boxplot figures in the Results section of the main paper (Figures 2–7) and is supplied here to guarantee complete transparency and reproducibility of statistical findings.

**Section S1 – Cytokines**

**Table S1.** Wilcoxon Rank Sum test for IL-10 and IL-6 concentrations in NCC patients and controls.

| | **Wilcoxon Scores (ranked Sum)** | | | | | **Wilcoxon Two-Sample test** | | |
|---|---|---|---|---|---|---|---|---|
| | N | Sum of Scores | Expected | Std dev | Mean score | Statistic | Z | *p*-value |
| IL-10 | | | | | | | | |
| **Control** | 11 | 66.000 | 126.500 | 15.229 | 6.000 | 66.000 | -3.940 | <.0001§ |
| **Patient** | 11 | 66.000 | 126.500 | 15.229 | 17.000 | 66.000 | -3.940 | <.0001§ |
| IL-6 | | | | | | | | |
| **Control** | 11 | 66.000 | 126.500 | 15.229 | 6.000 | 66.000 | -3.940 | <.0001§ |
| **Patient** | 11 | 66.000 | 126.500 | 15.229 | 17.000 | 66.000 | -3.940 | <.0001§ |

## Section S2- Hormones

**Table S2.** Wilcoxon Rank Sum test for Progesterone (P4) comparison for controls and patients

| | Wilcoxon Scores (ranked sum) | | | | | Wilcoxon Two-Sample test | | |
|---|---|---|---|---|---|---|---|---|
| | N | Sum of Scores | Expecte d | Std dev | Mean score | Statistic | Z | *p* -value |
| P4 | | | | | | | | |
| **Control** | 11 | 84.0 | 126.50 | 15.2288 | 7.6363 | 84.0000 | -2.7579 | 0.0058[§] |
| **Patient** | 9 | 169.0 | 126.50 | 15.2288 | 15.3636 | 84.0000 | -2.7579 | 0.0058[§] |

**Table S3.** Wilcoxon Rank Sum test for Androstenedione (A4) comparison for controls and patients

| | Wilcoxon Scores (ranked Sum) | | | | | Wilcoxon Two-Sample Test | | |
|---|---|---|---|---|---|---|---|---|
| A4 | N | Sum of Scores | Expected | Std dev | Mean score | Statistic | Z | *p*-value |
| **Control** | 11 | 66.0 | 126.50 | 15.2288 | 6.0 | 66.000 | -3.9399 | < 0.0001[§] |
| **Patient** | 11 | 187.0 | 126.50 | 15.2288 | 17.0 | 66.000 | -3.9399 | < 0.0001[§] |

**Table S4.** Wilcoxon Rank Sum test for Dihydrotestosterone (DHT) comparison for controls and patients

| | Wilcoxon Scores (ranked Sum) | | | | | Wilcoxon Two-Sample Test | | |
|---|---|---|---|---|---|---|---|---|
| | N | Sum of Scores | Expected | Std dev | Mean score | Statistic | Z | *p*-value |
| DHT | | | | | | | | |
| **Control** | 11 | 187.0 | 126.50 | 15.2288 | 17.0 | 187.000 | 3.9399 | < 0.0001[§] |
| **Patient** | 11 | 66.0 | 126.50 | 15.2288 | 6.0 | 187.000 | 3.9399 | < 0.0001[§] |

**Table S5.** Wilcoxon Rank Sum test for Luteinizing Hormone (LH) and Follicle Stimulating Hormone (FSH) comparison for controls and patients

| | Wilcoxon Scores (ranked Sum) | | | | | Wilcoxon Two-Sample Test | | |
|---|---|---|---|---|---|---|---|---|
| | N | Sum of Scores | Expected | Std dev | Mean score | Statistic | Z | *p*-value |
| FSH | | | | | | | | |

| | | | | | | | | |
|---|---|---|---|---|---|---|---|---|
| **Control** | 11 | 66.0 | 126.50 | 15.2245 | 6.000 | 66.000 | -3.9410 | < 0.0001[§] |
| **Patient** | 11 | 187.0 | 126.50 | 15.2245 | 17.000 | 66.000 | -3.9410 | < 0.0001[§] |
| LH | | | | | | | | |
| **Control** | 11 | 88.0 | 126.50 | 15.2288 | 8.000 | 88.000 | -2.4953 | 0.0126[§] |
| **Patient** | 9 | 165.0 | 126.50 | 15.2288 | 15.000 | 88.000 | -2.4953 | 0.0126[§] |

**Table S6.** Wilcoxon Rank Sum test for Free Testosterone (FT) and Estradiol (E2) comparison for control and patients

| | **Wilcoxon Scores (ranked Sum)** | | | | | **Wilcoxon Two-Sample Test** | | |
|---|---|---|---|---|---|---|---|---|
| | N | Sum of Scores | Expecte<br>d | Std dev | Mean score | Statistic | Z | *p*-value |
| T4 | | | | | | | | |
| **Control** | 1<br>1 | 180.0 | 126.50 | 15.2288 | 16.3636 | 180.000 | 3.4802 | 0.0005[§] |
| **Patient** | 1<br>1 | 73.0 | 126.50 | 15.2288 | 6.6363 | 180.000 | 3.4802 | 0.0005[§] |
| E2 | | | | | | | | |
| **Control** | 1<br>1 | 66.0 | 126.50 | 15.229 | 6.000 | 66.000 | -3.940 | <.0001[§] |
| **Patient** | 1<br>1 | 187.0 | 126.50 | 15.229 | 17.000 | 66.000 | -3.940 | <.0001[§] |
